# In vitro characterization of cellular responses elicited by endosomal TLR agonists encapsulated in Qβ virus-like particles

**DOI:** 10.1186/s12865-025-00768-7

**Published:** 2025-11-17

**Authors:** M. M. Hasibuzzaman, Briana Ibarra, Ishrat Nourin Khan, Thomas Craig Meagher, Caitlin D. Lemke-Miltner, George J. Weiner, Andrean L. Simons

**Affiliations:** 1 Interdisciplinary Graduate Program in Human Toxicology, University of Iowa, Iowa City, IA USA; 2 Department of Radiation Oncology, University of Iowa Hospitals and Clinics, Iowa City, IA USA; 3 Holden Comprehensive Cancer Center, University of Iowa Hospitals and Clinics, Iowa City, IA USA; 4 San Jacinto College, Houston, TX USA; 5 Regeneron Pharmaceuticals, Tarrytown, NY USA; 6 Department of Internal Medicine, University of Iowa Hospitals and Clinics, Iowa City, IA USA

**Keywords:** Vidutolimod, TLR, DCs, T cells, CD4, CD8

## Abstract

**Background:**

Despite promising clinical data for Toll-like receptor-9 agonists encapsulated in virus-like particles (TLR9a VLPs), the relative potency and mechanisms of TLR7a and dual TLR7/8a VLPs remain undefined. TLR9a VLPs, also known as Vidutolimod or CMP-001 is a novel TLR9a encapsulated in Qβ VLPs, which can activate plasmacytoid DCs (pDCs) and promote T cell activation. Other endosomal TLRs such as TLR7 and TLR8 expressed in DCs have been studied in several preclinical and clinical studies; however, their immune-activating properties when encapsulated in VLPs have not been tested before. Here, we utilized a series of in vitro experiments to test and compare immune cell activation stimulated by agonists to TLR7 (TLR7a), TLR7/8 (TLR7/8a), and TLR9 (TLR9a) when encapsulated in Qβ VLPs.

**Methods:**

Activation of immune cells (monocytes, natural killer (NK) cells, T cells, pDCs, and monocytic DCs (mDCs)) in response to TLR7a, TLR7/8a and TLR9a VLPs, was evaluated using flow cytometry, intracellular cytokine staining (ICS) and ELISA. Neutralizing cytokine antibodies, immune cell depletion kits and transwell models were used to determine the contribution of select cytokines and antigen presenting cells (APCs) in VLP-mediated immune cell activation.

**Results:**

Results showed that all three VLPs activated pDCs and monocytes. However, TLR7/8a VLPs were most effective at NK and T cell activation compared to the other VLPs. NK cells were a major source of IFNγ, whereas pDCs were the main source of IFNα and TNFα production in response to the VLPs. Neutralizing antibodies against TNFα (but not IFNα) showed significant suppressive effects on TLR7/8a VLP-mediated activation of CD4 + and CD8 + T cells. Depletion of APCs completely abrogated TLR7/8 VLP-mediated activation of CD4 + and CD8 + T cells. Lastly, TLR7/8a VLP-mediated activation of T cells was highly dependent on direct contact with pDCs (and not DC1 and DC2 subsets).

**Conclusions:**

In summary, endosomal TLRa VLPs all have the ability to activate pDCs, however, combined TLR7/8 activation using TLR7/8a VLPs was significantly more effective than the other VLPs at activating T cells and was dependent on direct contact with pDCs. Therefore, TLR7/8a VLPs may potentially induce a robust anti-tumor immune response and warrant further investigation for cancer therapy.

## Background

Dendritic cells (DCs) play a critical role in the ability of the immune system to recognize and combat tumor cells. DCs are involved in a continuous interplay between innate and adaptive immunity. For example, in innate immune sensing, DCs produce and secrete cytokines, chemokines and growth factors in response to different types of danger signals release by tumor cells [1]. In adaptive immunity, DCs are critical players in the generation of tumor-specific T cells by capturing tumor antigens that are released into the tumor microenvironment (TME) and presenting these tumor antigens to naïve T cells [2]. However, in cancer patients, pre-existing DC numbers and activity have been shown to be limited in the TME, which impedes upon the orchestration of tumor-specific T cell responses. Additionally, tumors are often accompanied by an immunosuppressive environment that interferes with the proper function of DCs. Therefore, strategies that promote DC activity may improve anti-tumor immune responses.

Immature DCs are activated via pattern recognition receptors (PRRs) by recognizing danger signals such as pathogen-associated molecular patterns (PAMPs) or damage-associated molecular patterns (DAMPs) released by tumor cells [3]. PRRs include Toll-like receptors (TLRs) which recognize these danger signals and initiate NF-κB, IRF3/7 and/or inflammasome signaling pathways resulting in the production of proinflammatory cytokines, particularly type I IFNs. Type I IFNs are highly important for the triggering of antigen retention and presentation by DCs to enhance tumor antigen-specific CD8 + T-cell responses [4, 5]. Of the 10 TLRs found in humans, TLR7, TLR8 and TLR9 are widely expressed on the endosomal compartment of DCs, although expression of these TLRs varies among different subsets of DCs. For example, myeloid DCs (mDCs) express TLR8 but not TLR7 and TLR9 [6–8]; and plasmacytoid DCs (pDCs) express both TLR7 and TLR9 [8, 9]. TLR7 and TLR8 recognize single strand RNA (ssRNA) molecules; whereas TLR9 recognizes unmethylated CpG DNA [6, 10]. Upon TLR recognition of these nucleotides, they signal through a myeloid differentiation factor 88 (MyD88)-dependent pathway leading to the production of Type I IFNs [11].

Engagement of TLR7-9 in DCs leads to the production of unique patterns of cytokines and inflammatory mediators, resulting in diverse immune response profiles. pDCs produce large amounts of IFNα in response to TLR7 activation [9, 12] whereas mDCs produce mainly tumor necrosis factor-alpha (TNFα) and IL-12 after TLR8 activation [13, 14]. TLR9 engagement on pDCs display unique features depending on the presence of early vs. late endosomes. TLR9 activation in early endosomes result in mainly IFNα secretion and activation of innate immunity, whereas TLR9 activation in late endosomes results in interleukin-6 (IL-6) and TNFα secretion generating adaptive immune response [15]. Nevertheless, it is clear that both pDCs and mDCs are significant presenters of tumor antigens to T cells [16].

Stimulation of TLRs in DCs are currently being investigated as alternates and adjuvants to standard cancer immunotherapies in various clinical and preclinical studies [6, 17–25]. Various formulations of endosomal TLR agonists (TLRa) have been investigated but none of the endosomal TLRa have been FDA approved for cancer therapy with exception of FDA approval of the small molecule TLR7a imiquimod for superficial basal cell carcinoma [26]. We have previously reported that the TLR9a VLPs (vidutolimod), triggered the activation of pDCs which increased T cell infiltration to the draining lymph nodes, and significantly enhanced anti-tumor immune response to anti-PD-1 therapy in HNSCC tumors [20]. Currently TLR9a VLPs in combination with anti-PD-1 therapy is under investigation in clinical trials for melanoma and lymphoma patients (NCT02680184, NCT03084640, NCT03983668, NCT03618641). TLR9a VLPs consists of a CpG-A oligodeoxynucleotides (ODN) encapsulated viral-like particles (VLPs). The VLPs used for the encapsulation are non-replicating, purified coat protein from bacteriophage Qβ, which are deficient in endogenous Qβ genetic materials. The viral encapsulation has two distinct advantages over soluble unencapsulated agonists. First, VLP encapsulation protects CpG ODNs from premature destruction by endonuclease before it is taken up by APCs [27, 28]; and second, the viral protein may provide additional danger signals and alter uptake by antigen-presenting cells, including pDCs [29]. The efficacy of agents encapsulated by Qβ-derived VLPs such as TLR9a VLPs are dependent on B cell-derived anti-Qβ antibody production which allows for antibody-mediated opsonization of TLR9a VLPs via Fc receptors, leading to the uptake by pDCs and subsequent production of IFNα [12, 20].

In this current study we now begin to characterize using in vitro methods, two additional proprietary Qβ derived VLP-encapsulated endosomal TLRas: TLR7a VLPs which contain ssRNA against TLR7; and dual TLR7/8a VLPs which contain ssRNAs against TLR7 and TLR8. Here we show that TLR9a, TLR7a, and TLR7/8 VLPs all have the ability to activate pDCs, however, combined TLR7/8 activation using TLR7/8 VLPs was significantly more effective than the other VLPs at activating CD4 + and CD8 + T cells which was dependent on direct contact with pDCs.

## Materials and methods

### VLPs containing TLR7, TLR7/8 and TLR9 agonists, anti-Qβ antibody, and other reagents

 TLR9a VLPs (CMP-001/Viutolimod), TLR7a VLPs, TLR7/8a VLPs, EMPTY VLPs, recombinant anti-Qβ antibodies, and succinate buffer were provided by Regeneron Pharmaceuticals (Westchester, New York USA). All TLRa VLPs were manufactured using the bacteriophage Qβ nanotechnology platform. In this nanotechnology platform, nanoparticles were self-assembled upon mixing purified Qβ coat protein with CPG-A ODN (TLR9a VLP) or ssRNA (TLR7a VLP and TLR7/8a VLP) [30, 31]. The diameter of the resulting VLPs were between 25 and 30 nm. The TLRa VLPs used in this study were highly pure (less than 0.08 endotoxin units [EU]/mg) with similar particle size. The TLR7/8 agonist resiquimod (R848) was purchased from InvivoGen (Cat# tlrl-r848-1).

### Isolation of unfractionated human peripheral blood mononuclear cells (PBMCs)

Human peripheral blood mononuclear cells (PBMCs) were collected from healthy adult donor blood (*n* = 3–4, obtained from DeGowin Blood Center at the University of Iowa Hospitals and Clinics) by density gradient centrifugation using Ficoll paque density gradient media (Cytiva, Cat #17144003). Blood in 10 mL blood cones was first diluted in 20 mL cold phosphate-buffered saline (PBS) into a 50 mL conical tube. PBS containing blood sera were removed from the top and the remaining blood was resuspended in equal volumes of cold PBS. PBMCs were then separated using Ficoll gradient, PBMC buffy coats collected then transferred into new 50 mL conical tubes. After washing with excess PBS (up to 50 mL), the supernatant was removed and the PBMCs were resuspended into Roswell Park Memorial Institute (RPMI) 1640 medium supplemented with 10% heat-inactivated (56 °C, 30 min) fetal bovine serum (FBS). The volume was raised to 50 mL with PBS and tubes were spun at 400×g for 10 min at room temperature.

### Cytokine neutralization experiments

For cytokine neutralization experiments, fresh PBMCs were isolated and 500,000 cells plated in 96-well plates in 250 uL RPMI media. PBMCs were then treated with 10 µg/mL TLR7/8a VLPs plus 10 µg/mL anti-Qβ with or without anti-human IFNα (20 µg/mL, InvivoGen, Cat# hifna-mab1-02), TNFα (20 µg/mL, InvivoGen, Cat# htnfa-mab1) or IFNα + TNFα for 24 h, collected, washed then stained for flow cytometry analysis. Human IgG1 (20 µg/mL, InvivoGen, Cat# bgal-mab1) and succinate buffer were used as controls. Culture media was used to measure cytokine levels by ELISA.

### Isolation and depletion of immune cell subsets from unfractionated PBMCs

Immune cell subsets were depleted from freshly isolated PBMCs using magnetic coated microbeads according to the manufacturer’s protocols. Natural killer (NK) cells were depleted using anti-human CD56 MicroBeads (Miltenyi Biotec, #130-097-042), monocytes were depleted using anti-human CD14 MicroBeads (Miltenyi Biotec, # 130-050-201) and antigen presenting cells (APC) were depleted using anti-HLA-DR MicroBeads (Miltenyi Biotec, #130-046-101). All the described microbeads positively select specific immune cells from unfractionated PBMCs which are retained into the magnetic column, leaving untouched PBMCs populations depleted of specific immune cell subsets. Unfractionated and depleted PBMCs were then cultured in 96-well plates and treated with 5 µg/mL TLRa VLPs plus anti-Qβ for flow cytometric analyses. To observe the direct and indirect effect of dual TLR7/8a VLPs, T cells and different subsets of dendritic cells (DCs) were isolated from freshly collected PBMCs. T cells and DCs were donor matched for each individual experiment. T cells were first enriched from whole PBMCs using MojoSort™ Human CD3 T Cell Isolation Kit (BioLegend, #480022) through negative selection of the T cell population according to manufacturer’s protocol. Briefly, unfractionated PBMCs were resuspended in Mojoshrt Buffer (BioLegend, #480017) then incubated with a cocktail of antibody-conjugated magnetic beads. Magnetically labeled cells were then placed on a MojoSort™ Magnet (BioLegend, #480019). After incubation, untouched T cells were collected. T cell enriched PBMCs were then washed in FACS buffer and stained with a cocktail of fluorochrome-conjugated antibodies for FACS for purified T cells (CD45 + CD3 + CD19-, MHCII-). DCs were also first enriched from whole PBMCs using a human pan-DC Enrichment Kit (Miltenyi Biotec, #130-100-777) through negative selection according to the manufacturer’s protocol. DC-enriched PBMCs were then stained with a cocktail of fluorochrome-conjugated antibodies for FACS cell shorting. From the enriched DC population, pDCs were isolated using gating on live cells, CD45+, Lineage-, MHCII + CD11c-, CD304+, CD123+; CD1Cs were isolated using gating on live cell, CD45+, Lineage-, MHCII + CD11c+, CD1C+, CD123-,CD141-; and CDC2s were isolated using gating on live cell, CD45+, Lineage-, MHCII + CD11c+, CD1C-, CD123-,CD141+. Flow cytometry was used to confirm purity of cell fractions and the purity of both T cell and DC subsets were found to be more than 99% in this procedure.

### Immune cell co-cultures and transwell assays

Human CD3 + T cells and subsets of DCs (pDC, CD1c and CDC2) were isolated as described above. For co-culture experiments, pDCs, CD1Cs and CDC2s were co-cultured with isolated T cells. The T cells and DCs were co-cultured at a 50 (T cells):1(DCs) ratio in a 96 well flat-bottomed tissue culture plate in 250 uL RPMI media. This co-culture ratio was selected as the T cell population typically presents in higher number (45–70%) compared to DCs (1–2%) in whole PBMCs. T cell: DC co-cultures were then treated with TLR7/8a VLPs (5 µg/mL) plus anti-Qβ (5 µg/mL) and analyzed through flow cytometry for CD69 surface expression on CD4 + and CD8 + T cells. Cells were also treated with succinate buffer, PBS and EMPTY VLPs (plus anti-Qβ) as controls. For transwell experiments, pDCs and T cells were separated by a 0.4 μm pore polycarbonate membrane inserts (Corning, #3413). The membrane was pre-soaked with RPMI media and pDCs were plated on top of the membrane in 100 uL media. On the bottom chamber T cells were plated in 600 uL RPMI media. pDCs on the top chamber were then treated with TLR7/8a VLPs plus anti-Qβ. In the absence of transwells, pDCs and T cells were co-cultured under identical conditions and used as a positive control.

### ELISA Assay

Levels of IFNα, TNFα and IFNγ were measured in the cell culture media of PBMCs using enzyme-linked immunosorbent assay (ELISA). TNFα (Cat# DY210) and IFNγ (Cat#DY285B) were detected though Human Duo Set ELISA kits according to the manufacturer’s protocol (R&D Systems, Minneapolis, MN). Levels of IFNα were measured through Human IFN-Alpha Multi-Subtype ELISA Kit from PBL Assay Science (Cat# 41105). A Synergy H1 Hybrid Multi-Mode Reader (BioTek, Winooski, VT) was used for colorimetric analysis.

### Flow cytometry

 Unfractionated and depleted PBMCs, isolated T cells and DCs (pDC, CD1c, CDC2) were isolated and treated as indicated. After incubation with specific VLPs and control drugs, cells were harvested and washed with PBS for staining with Zombie Aqua Viability Dye (Biolegend #423102). Cells were then washed with FACS buffer and stained with cocktail of antibodies for flow cytometry analysis of different immune cell subset and their activation. CD4 + T cells were defined as CD45 + CD3 + CD4+; CD8 + T cells were defined as CD45 + CD3 + CD8+; NK cells were defined as CD45 + CD3- CD19- MHC II- CD56+; monocytes were defined as CD45 + CD3- CD19- MHC II + CD14+; pDCs were defined as CD45 + CD3- CD19- CD14- CD11c- BDCA-4 + CD123+; and mDCs were defined as CD45 + CD3- CD19- CD14- CD11c + CD123-. T and NK cell activation were determined by CD69 expression on these cells, whereas monocyte and pDC activation were determined as CD40 expression on these cells. For intracellular cytokine staining, unfractionated and depleted PBMCs were treated with 10 µg/mL TLRa VLPs plus anti-Qβ for 2 h then treated with brefeldin A (BFA) (Biolegend #420601) solution to enhance intracellular cytokine staining signals. Following another 16–18 h of incubation, cells were then harvested and stained with Zombie Aqua Viability Dye and a cocktail of surface antibodies as described above. After surface staining, cells were fixed and permeabilized with eBioscience Intracellular Fixation and Permeabilization Buffer (Thermo Fisher Scientific #88–8824-00). Intracellular staining was then performed by staining with antibodies against IFNα, TNFα and IFNγ (Miltenyi Biotec #130-104-963). All samples were fixed on 2% Paraformaldehyde Solution (PFA) (Fisher Scientific, Cat#AAJ19943K2) after staining and assessed within 24 h on a Cytek Aurora five laser flow cytometer. Data were analyzed using FlowJo V.10.2.

### Statistical analyses

Cytokine data and immune cell activation by flow cytometry data were analyzed by one-way ANOVA with Tukey’s multiple comparisons test. Cytokine blockade data were also evaluated by one-way ANOVA with Tukey’s multiple comparisons test. Immune cell isolation and blockade data were analyzed by unpaired Student’s t-test and one-way ANOVA with Tukey’s multiple comparisons test, respectively. Finally, transwell assay data were analyzed by unpaired Student’s t-test. All the statistical analysis was carried out using GraphPad Prism V.8 for Windows (GraphPad Software, San Diego, California, USA). A p value of < 0.05 was considered significant.

## Results

### Endosomal TLRa VLPs trigger the release of both IFNα and TNFα from human PBMCs

**Fig. 1:**
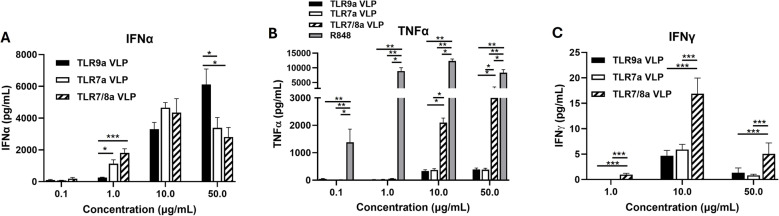
Endosomal TLRa VLPs trigger the release of both IFNα and TNFα from human PBMCs. Peripheral blood mononuclear cells (PBMCs) were treated with escalating doses (0.1–50 µg/mL) of virus-like particles (VLPs) encapsulating TLR7, TLR9 and TLR7/8 agonists (in addition to identical doses of anti-Qβ) for 24 h then analyzed for interferon alpha (IFNα) **A**, tumor necrosis factor alpha (TNFα) **B**, and interferon gamma (IFNγ) **C** in cell culture media by ELISA. Bars represent the mean of *n* = 3 biological replicates. Error bars represent SE from the mean. *p <.05; **p <.01; ***p <.001

 To characterize the immunostimulatory activity of the TLRa VLPs, freshly isolated human PBMCs were treated with succinate (control) and escalating doses (0.1–50 µg/mL) of TLR7a, TLR9a and TLR7/8a VLPs (in addition to identical doses of anti-Qβ) for 24 h then analyzed for IFNα, TNFα, and IFNγ in cell culture media by ELISA. All the tested TLRa VLPs triggered IFNα secretion at 1, 10 and 50 µg/mL with TLR7a and TLR7/8a VLPs triggering significantly higher levels of IFNα at 1 µg/mL compared to TLR9a VLPs; and TLR9a VLPs triggering significantly higher IFNα levels at 50 µg/mL compared to TLR7a and TLR7/8a VLPs (Fig. 1A). While TNFα was secreted from PBMCs in response to R848 (positive control) at all doses and all 3 TLR VLPs (at 10 and 50 µg/mL), TLR7/8a VLPs triggered significantly more TNFα secretion at 10 and 50 µg/mL than TLR7a and TLR9a VLPs (Fig. 1B). TLR7/8a VLPs triggered IFNγ secretion at all doses with the most robust secretion at 10 µg/mL (Fig. 1C**).** TLR9a and TLR7a VLPs also triggered IFNγ secretion at all doses but significantly less so than TLR7/8a VLPs (Fig. 1C). Succinate controls did not trigger any cytokine (IFNα, TNFα, IFNγ) production are not shown. Together these results suggest that all 3 TLR VLPs have similar effects on IFNα secretion, but dual activation of TLR7 and TLR8 using the TLR7/8a VLPs are most effective at the production of TNFα and IFNγ compared to TLR9a and TLR7a VLPs.

### Endosomal TLRa VLPs activate immune cells

**Fig. 2:**
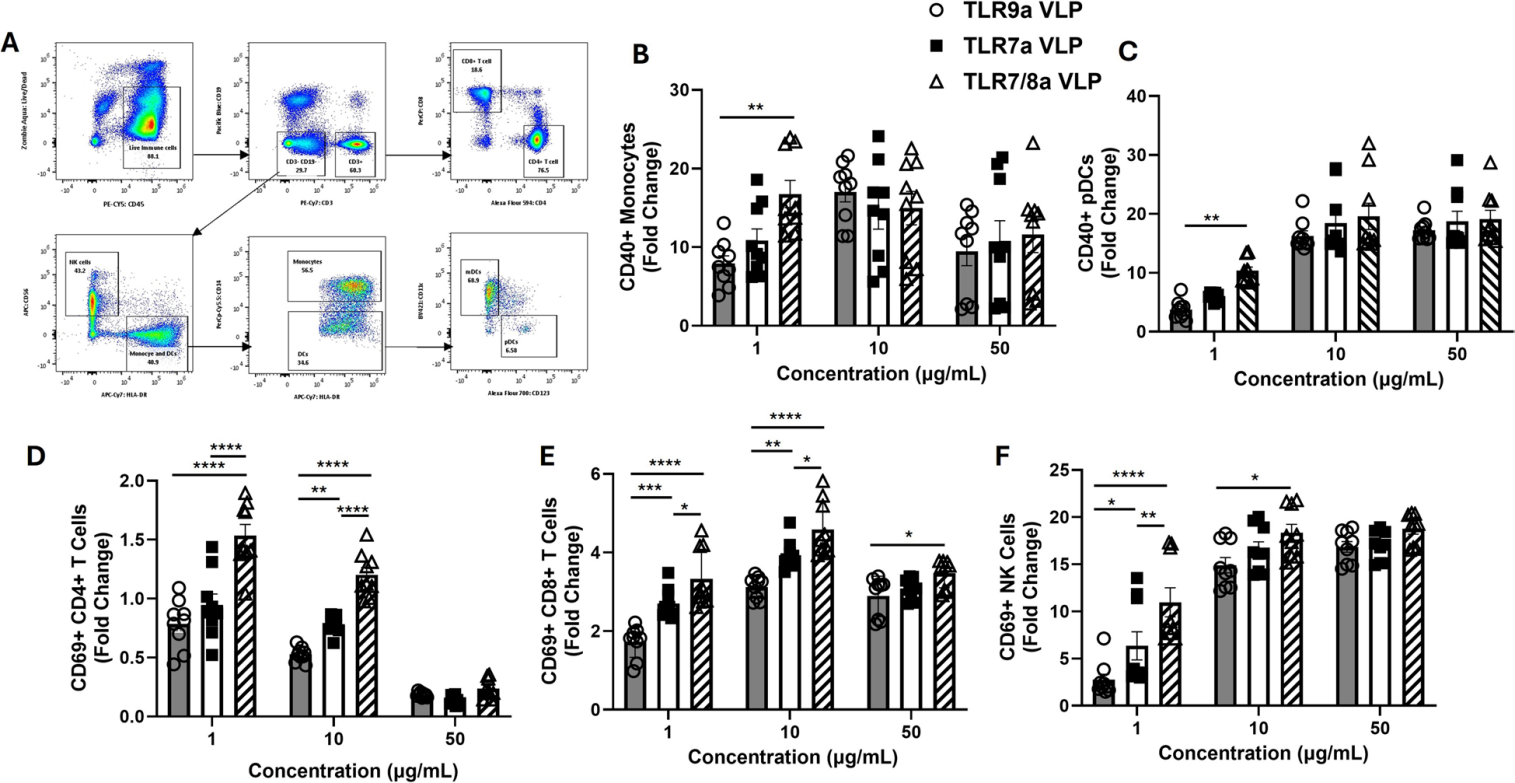
Endosomal TLRa VLPs activate immune cells. Peripheral blood mononuclear cells (PBMCs) were treated with escalating doses (1–50 µg/mL) of virus-like particles (VLPs) encapsulating TLR7, TLR9 and TLR7/8 agonists (in addition to identical doses of anti-Qβ) for 24 h then analyzed by flow cytometry for the indicated activated immune cells. **A** Flow cytometry gating strategy to identify CD40 + monocytes (antibody-dependent, cell-mediated cytotoxicity), and plasmacytoid dendritic cells (pDCs) **B** and CD69 + CD4 + T cells (**D**), CD8 + T cells **E** and natural killer (NK) cells **F** from human PBMCs. Average values were normalized to succinate control and plotted as fold change. Bars represent the mean of *n* = 3 biological replicates. Error bars represent SE from the mean. *p <.05; **p <.01; ***p <.001; ****p <.0001

We next investigated the ability of the TLR VLPs to stimulate innate and adaptive immune cells in freshly isolated human PBMC cultures. The gating strategy is shown in Fig. 2A. CD69 surface expression was used to detect activated T and NK cells; and CD40 surface expression was used to detect monocyte and pDC activation. All 3 TLR VLPs activated monocytes **(**Fig. 2B**)** and pDCs **(**Fig. 2C**)** to a similar extent at 10 and 50 µg/mL compared to succinate control (set at fold change = 1), with TLR7/8a VLPs being most effective at 1 ug/ml. Activation of CD4 + T cells was observed only with TLR7/8 VLPs at 1 and 10 ug/ml (Fig. 2D), however activation of CD8 + T cells (Fig. 2E) and NK cells (Fig. 2F) was observed with all the TLR VLPs with TLR7/8a VLPs being most effective at 1 and 10 ug/ml. Together, these results suggest that all 3 TLR VLPs have the ability to activate monocytes, pDCs, NK and T cells, although dual activation of TLR7 and TLR8 using the TLR7/8a VLPs are most effective at NK and T cell activation compared to TLR9a and TLR7a VLPs.

### Endosomal TLRa VLPs induce the production of cytokines in immune cells

**Fig. 3:**
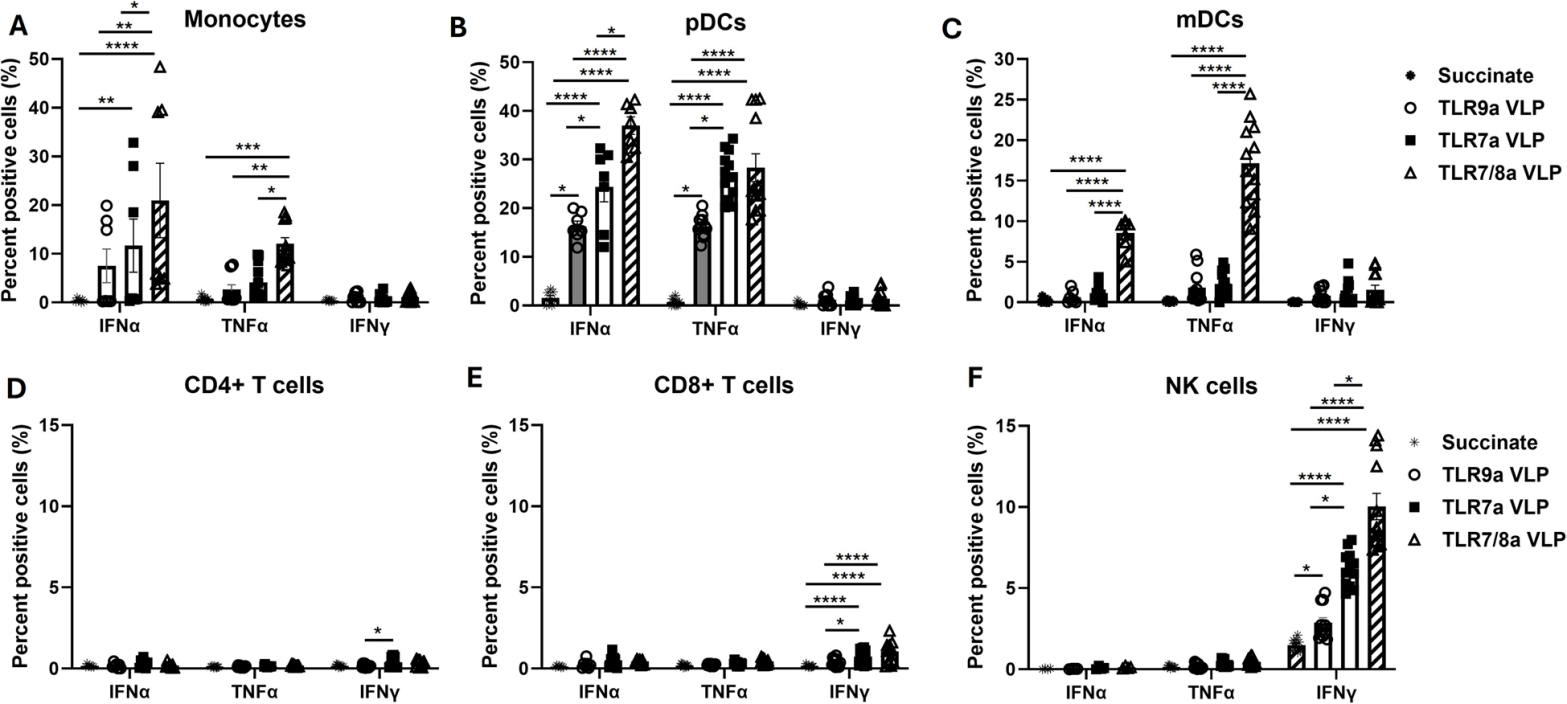
Endosomal TLRa VLPs induce the production of cytokines in immune cells. Freshly isolated peripheral blood mononuclear cells (PBMCs) were stimulated by the indicated VLPs (10 µg/mL) plus anti-Qβ (10 µg/mL) for 2 h then treated with brefeldin A for 16–18 h. Surface and intracellular staining was performed to identify IFNα, TNFα and IFNγ positive monocytes **A** plasmacytoid dendritic cells (pDCs) **B** monocytic DCs **C** CD4 + T cells **D** CD8 + T cells **E**, ad natural killer (NK) cells **F** by flow cytometry analyses. Succinate buffer was used as a control. Error bars represent SE from the mean. Bars represent the mean of *n* = 3 biological replicates. *p <.05; **p <.01; ***p <.001; ****p <.0001

To elucidate the cell subsets in PBMCs that were the source of effector cytokines stimulated by TLRa VLPs, intracellular staining followed by flow cytometry was carried out on PBMCs. Brefeldin A was used following a short-term activation period (2 h) which blocks cytokine release from other accessory cells. TLR7/8a VLP stimulation produced significantly higher percentages of IFNα and TNFα-positive monocytes (Fig. 3A), pDCs (Fig. 3B) and mDCs (Fig. 3C) compared to TLR7a and TLR9a VLP stimulation. TLR7a and TLR9a VLP stimulation produced significantly higher percentages of IFNα and TNFα-positive pDCs (but not mDCs) compared to succinate control (Fig. 3B, C). TLR7a VLPs also stimulated IFNα-positive monocytes compared to control (Fig. 3A). None of the TLRa VLPs stimulated monocytes, pDCs or mDCs to produce IFNγ production (Fig. 3A-C). These data suggest that APCs such as monocytes, pDCs and mDCs are major sources of IFNα and TNFα production by TLRa VLPs. Moreover, it appears that TLR7a and TLR9a VLPs stimulates pDCs only, while TLR7/8a VLPs stimulate both pDCs and mDCs. TLR9a VLPs did not trigger any IFNα, TNFα or IFNγ-positive T cells (Fig. 3D, E). However, TLR7a and TLR7/8a VLPs resulted in the minimal but significant increase in IFNγ-positive CD8 + T cells compared to succinate control (Fig. 3E). All VLPs triggered a significant increase in IFNγ-positive NK cells compared to succinate control with TLR7/8a VLPs being the most effective compared to the other 2 VLPs (Fig. 3F). Together, these results indicate that TLRa VLPs induce minimal changes in cytokine-positive T cells, but robustly stimulate the production of IFNγ-positive NK cells with TLR7/8a being the most effective among the TLRa VLPs.

### TNFα partially mediates TLR7/8a VLP-induced activation of T cells

**Fig. 4:**
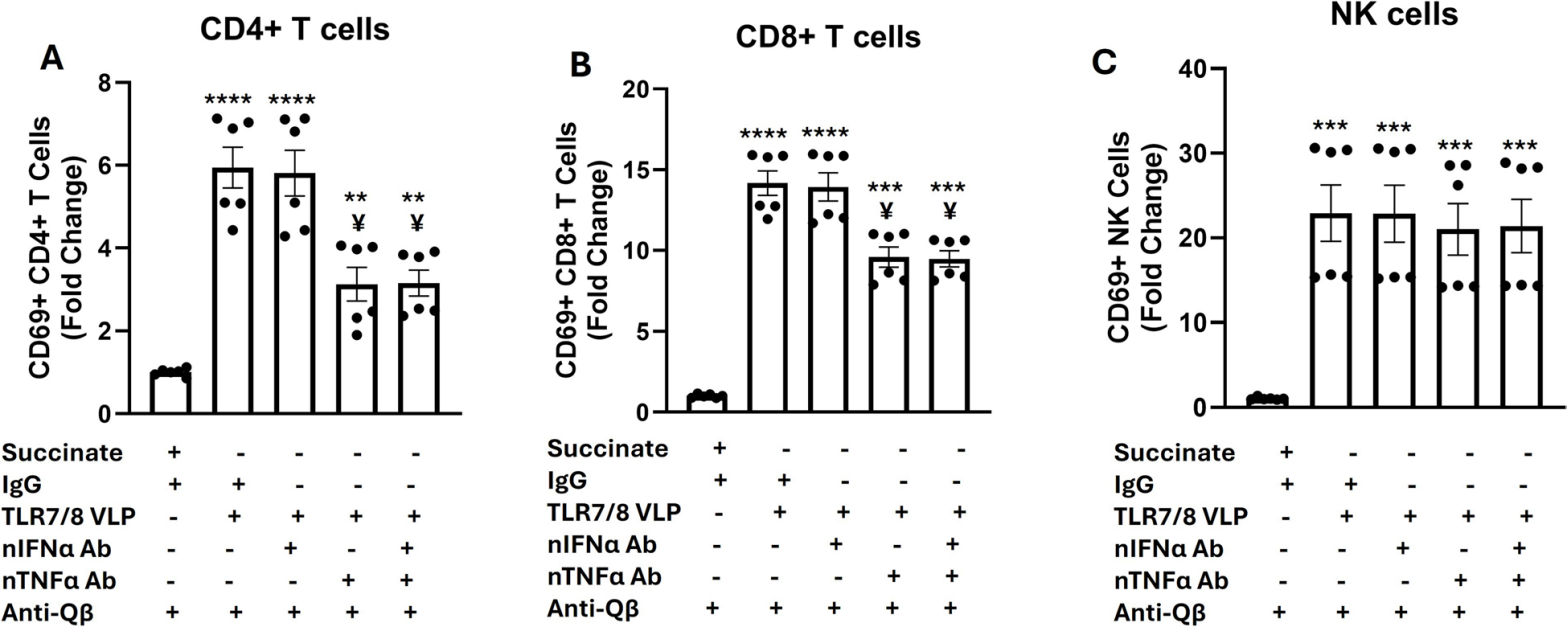
TNFα partially mediates TLR7/8a VLP-induced activation of T cells. Freshly isolated peripheral blood mononuclear cells were treated with TLR7/8a VLPs (10 µg/mL) plus anti-Qβ (10 µg/mL) with and without neutralizing antibodies (20 µg/mL) to IFNα (nIFNα Ab), TNFα (nTNFα Ab), and nIFNα Ab + nTNFα Ab for 24 h. IgG and succinate buffer were used as controls. Cells were then harvested and analyzed by flow cytometry for CD4 + T cell **A **CD8 + T cell **B** and NK cell **C** activation. Error bars represent SE from the mean. Bars represent the mean of *n* = 3 biological replicates. **, ***, and **** indicates *p* <.01, *p* <.001, and *p* <.0001 versus Succinate. ¥ indicates *p* <.05 versus TLR7/8 VLP

 Because of the significant effects of TLR7/8a VLPs on T and NK cell activation (Fig. 2D-F) compared to the other VLPs we proceeded to focus on the effect of TLR7/8a VLPs for the remainder of the studies. To determine whether IFNα and/or TNFα play a role in the activation of T and NK cells by TLR7/8a VLPs, these cytokines were blocked with neutralizing IFNα and/or TNFα antibodies during the 24-hour treatment of PBMCs with TLR7/8a VLPs. Flow cytometry experiments were then carried out for CD69 surface expression on CD4 + T cells, CD8 + T cells and NK cells. Neutralization of IFNα did not suppress the activation of CD4+ (Fig. 4A) and CD8+ (Fig. 4B) T cells triggered by TLR7/8a VLPs. Neutralization of TNFα partially but significantly suppressed both CD4 + and CD8 + T cell activation (Fig. 4A, B) triggered by TLR7/8a VLPs. Combined neutralization of IFNα and TNFα did not suppress T cell activation (triggered by TLR7/8a VLPs) beyond that of IFN neutralization (Fig. 4A, B). Neither the blockade of IFNα, TNFα or their combination was able to suppress TLR7/8a VLP-mediated activation of NK cells (Fig. 4C). Altogether, TNF appears to play a role in TLR7/8a VLP-mediated activation of T cells however there are likely other cytokines or mediators involved.

### Dual TLR7/8a VLP mediated T cell activation is dependent on antigen presenting cells

**Fig. 5:**
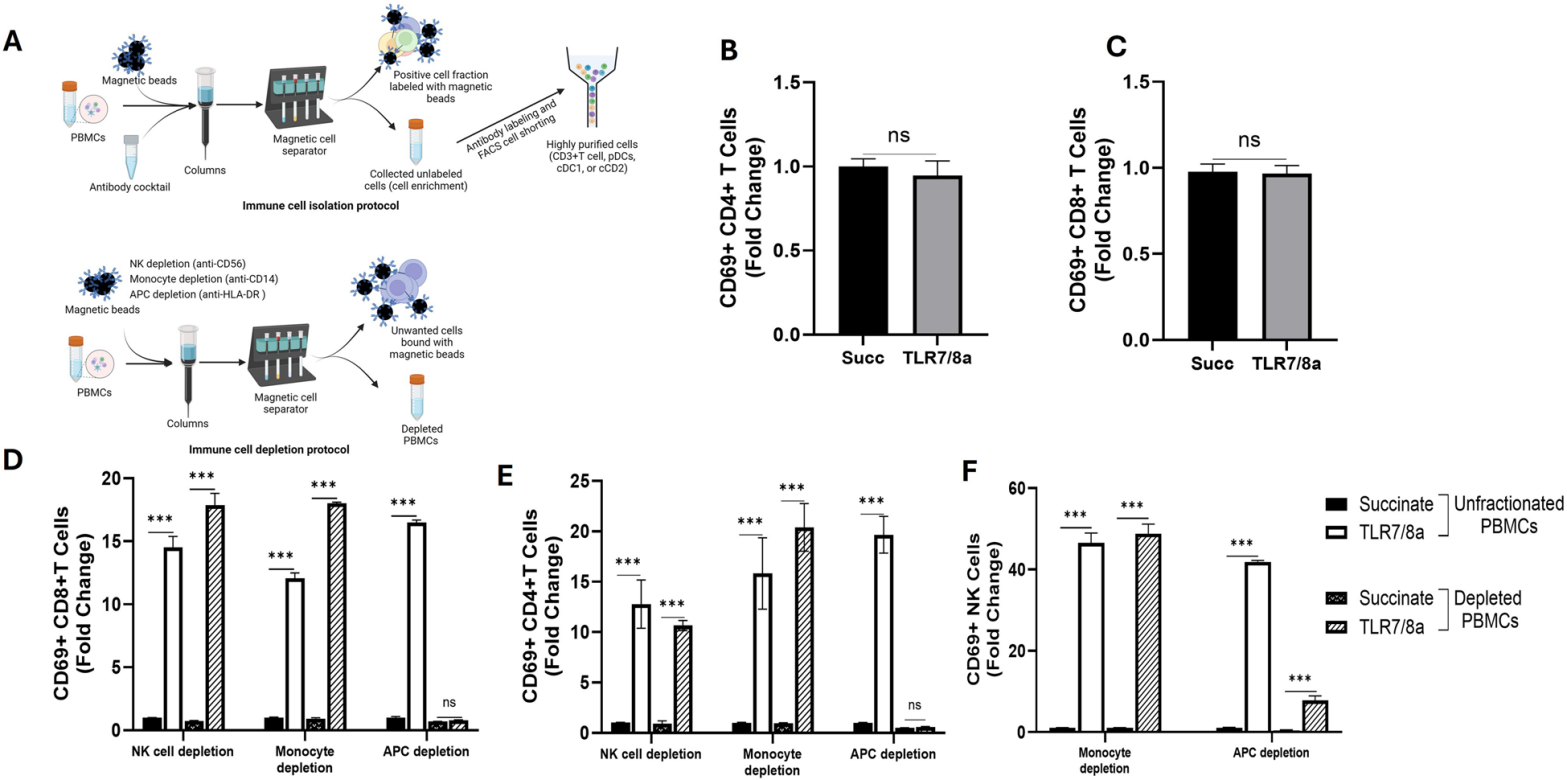
TLR7/8a VLP-mediated T cell activation is dependent on antigen presenting cells. T cells were isolated from unfractionated PBMCs (**A**) and treated with TLR7/8a VLPs (10 µg/mL) plus anti-Qβ (10 µg/mL) for 24 h then analyzed for CD69 surface expression on CD4+ **B** and CD8+ **C** T cells. Subsets of immune cells (NK cells, monocytes and APCs) were depleted from PBMCs **A** treated with TLR7/8a VLPs (10 µg/mL) plus anti-Qβ (10 µg/mL) for 24 h then analyzed for CD69 surface expression on CD4+ **D **and CD8+ **E** and NK cells **F**. Succinate buffer (Succ) was used as a control. Average values were normalized to succinate control and plotted as fold change. Error bars represent SE from the mean. Bars represent the mean of *n* = 3 biological replicates. ***p <.001; ns: non-significant

To determine if TLR7/8a VLPs directly activated T and NK cells. We collected highly purified (> 99%) CD3 + T cells from fresh PBMCs (Fig. 5A) and analyzed CD69 expression changes on both CD4 + and CD8 + T cells after stimulation with TLR7/8a VLPs. CD4+ (Fig. 5B) and CD8+ (Fig. 5C) T cells were not activated in response to TLR7/8a VLP stimulation suggesting there was no direct activation. To investigate the possible involvement of other immune cells responsible for T cell activation, we first depleted specific immune cell populations (NK cells, monocytes, APCs) from freshly isolated human PBMCs through negative selection (Fig. 5A) and the depleted PBMC populations were stimulated by TLR7/8a VLPs before analysis of changes in CD69 expression by flow cytometry. Results showed that NK cell and monocyte depletion did not suppress TLR7/8a VLP-mediated activation of CD4+ (Fig. 5D) and CD8+ (Fig. 5E) T cells. However, APC depletion completely abrogated TLR7/8a VLP-mediated activation suggesting indirect activation of T cells via APCs. Likewise, we found that monocyte depletion did not suppress TLR7/8a VLP-mediated NK cell activation, but APC depletion significantly (but not completely) suppressed NK cell activation (Fig. 5F) suggesting that NK cells may be stimulated by both direct and indirect mechanisms after TLR7/8a VLP treatment.

### Dual TLR7/8a VLP mediated activation of CD4 + and CD8 + T cells is dependent on pDC help

**Fig. 6:**
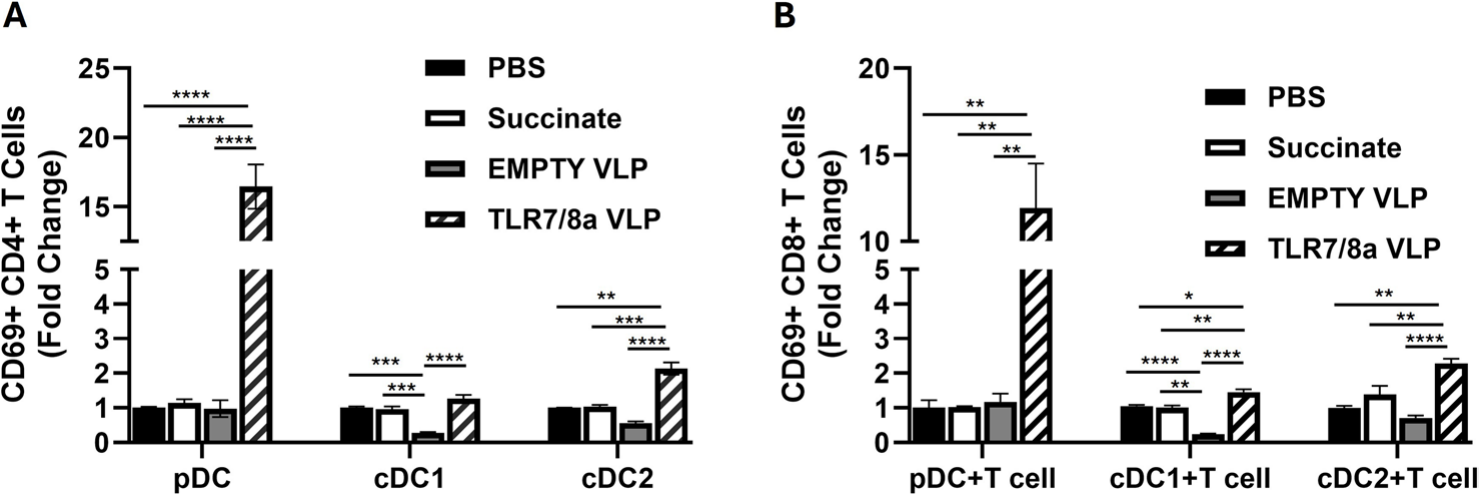
TLR7/8a VLP-mediated activation of T cells is dependent on pDC help. Subsets of dendritic cells (pDC, cDC1 and cDC2) were isolated from fresh PBMCs and co-cultured with freshly isolated T cells from the same donor. The DC: T cell co-cultures were treated with TLR7/8a VLPs (10 µg/mL) plus anti-Qβ (10 µg/mL) for 24 h and analyzed by flow cytometry for CD69 surface expression on CD4+ **B** and CD8 + T **C** cells. Succinate, PBS and EMPTY VLPs were used as controls. Average values were normalized to PBS control and plotted as fold change. Bars represent the mean of *n* = 3 biological replicates. *p <.05; **p <.01; ***p <.001; ****p <.0001

Given the prior observed effects of TLR7a VLPs on DCs (Fig. 3B, C) and their potential role in T cell activation (Fig. 5D, E), we further investigated which type of DC is required for priming and activation of T cells. We isolated highly pure subsets of DCs (pDC, CD1c and CDc2) and T cells from human PBMCs as described in Fig. 5A. Subsets of DCs and T cells were donor matched, co-cultured, then stimulated with TLR7/8a VLPs or control treatments (PBS, succinate and EMPTY VLPs). Results showed significantly higher activation of both CD4+ (Fig. 6A) and CD8+ (Fig. 6B) when co-cultured with pDCs compared to when co-cultured with cDC1 and cDC2 subsets. TLR7/8a VLPs did minimally (but significantly) activate CD4 + and CD8 + T cells when co-cultured with cDC1 and cDC2 subsets compared to EMPTY VLPs (Fig. 6A, B), however this activation but was not as robust as observed with pDCs. These data suggest that pDC help is important for TLR7/8a VLP-mediated activation of T cells.

### TLR7/8a VLP-mediated T cell activation through pDCs is contact dependent

**Fig. 7:**
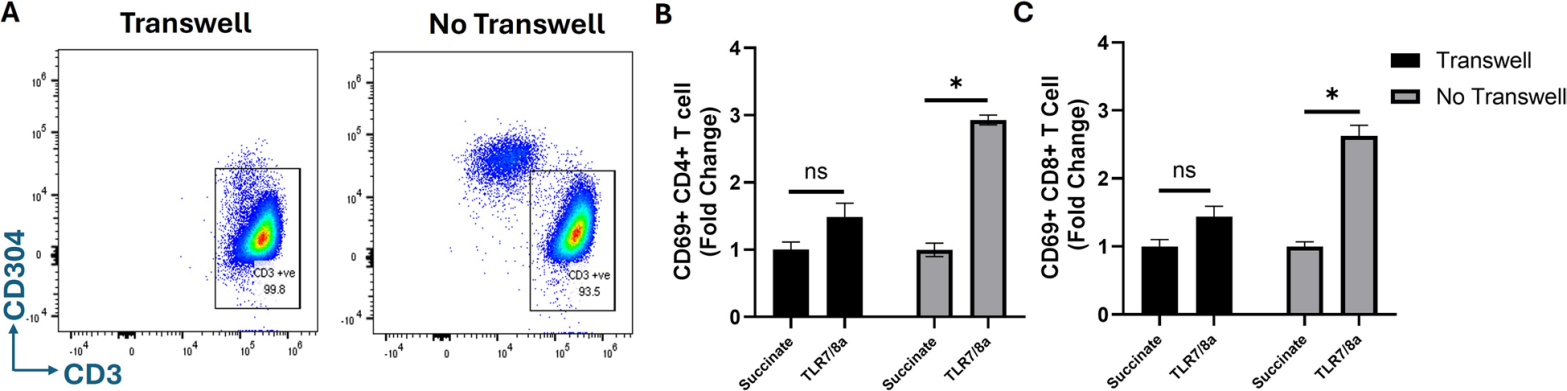
TLR7/8a VLP-mediated T cell activation through pDCs is contact dependent. Human T cells and pDCs were isolated from PBMCs then cultured in transwell plates where pDC and T cells were separated by a 0.4 μm pore polycarbonate membrane insert. pDCs were treated with TLR7/8a VLPs (10 µg/mL) plus anti-Qβ (10 µg/mL) for 24 h. Succinate was used as a control. Cells from the bottom chamber were stained for CD304 expression to check for any pDCs that migrated across the membrane **A**. T cells from the bottom chamber were collected and analyzed through flow cytometry for changes in CD69 surface expression on CD4+ **B** and CD8+ **C **T cells. Average values were normalized to succinate control and plotted as fold change. Error bars represent SE from the mean. *p <.01 versus succinate control; ns: non-significant

To determine if direct cell-to-cell contact is required for pDCs to induce T cell activity in response to TLR7/8a VLPs, pDCs were plated on the top of transwells containing 0.4 μm size pores. T cells were plated on the bottom of the transwell and pDCs on the top chamber were treated with TLR7/8a VLPs for 24 h. T cells from the bottom chamber were collected and evaluated by flow cytometry for changes in CD69 surface expression. Cells from the bottom chamber were stained for CD304 to confirm the absence of pDC migration across the membrane (Fig. 7A). Results indicated significant activation of both CD4+ (Fig. 7B) and CD8+ (Fig. 7C) T cells in the absence of transwells, but not the presence in transwells. This data suggest that direct contact is likely required between pDCs and T cells for robust TLR7/8a VLP-mediated T cell activation.

## Discussion

The results of this in vitro study point to TLR7/8a VLPs as a promising candidate for further preclinical studies given its observed superiority over the TLR9a and TLR7a VLPs at immune cell activation in studies with PBMCs (Figs. 2 and 3). The finding that the TLR7/8 VLPs are more effective at T cell activation than the TLR9a VLPs in particular is significant, since there are ongoing clinical investigations of this VLP (TLR9a VLPs/Vidutolimod) in the cancer-immunotherapy field due to positive results being obtained when combined with pembrolizumab in a phase 1b trial in anti-PD1-resistant metastatic melanoma patients (NCT02680184, Registration Date: 02/11/2016) [18], and combined with nivolumab in a phase II trial in stage IIIb/c/d melanoma patients with lymph node disease (NCT03618641) [25]. This has now led to an additional trial for this drug (combined with pembrolizumab) at the University of Iowa for lymphoma (NCT03983668). Our results raise the question of why dual activation of TLR7 and TLR8 is more effective in PBMCs at T cell activation (Fig. 2D, E) than TLR9 agonism despite all the VLPs being equally effective at increasing the number of activated pDCs and monocytes (Fig. 3B, C). Previous work using unencapsulated TLR7, TLR8 and TLR9 agonists support these findings in that agonists to TLR7 and TLR7/8 are more effective than TLR9 agonists at T cell activation despite similar effects on DC activation [32–38]. Our data suggests that this finding may be due to the broader activation of APCs by TLR7/8a VLPs, since monocytes, pDCs and mDCs, which are major sources of IFNα and TNFα, were stimulated be TLR7/8 VLPs; but only pDCs (and to a variable extent monocytes) were stimulated by TLR9a VLPs (Fig. 3A-C). Additionally, pDCs express TLR7 and TLR9 (but not TLR8), whereas mDCs express TLR8 (but not TLR7 and TLR9) [6–9]. Therefore, it makes sense that dual agonism of TLR7 and TLR8 would trigger more robust APC activation than TLR9 agonism alone. Regarding monocytes which reportedly express TLR7, TLR8 and TLR9 [39–41], we observed that all the TLRa VLPs increased the number of activated (CD40+) monocytes (Fig. 2B) and IFNα-positive monocytes (Fig. 3A) compared to control although this is unclear whether the activation is due to TLR agonism or Fc gamma receptor (FcγR)-binding of anti-Qβ-bound VLPs. As mentioned before, the efficacy of agents encapsulated by Qβ-derived VLPs are dependent on B cell-derived anti-Qβ antibody production which allows for antibody-mediated opsonization via FcγR, leading to the uptake by APCs and subsequent production of IFNα [12, 20]. We and others have previously shown that anti-Qβ antibody binding is necessary for uptake of TLR9a VLPs by immune cells and its in vitro and in vivo activity [12, 18, 20, 28] which is why our studies included anti-Qβ antibodies with all VLP treatments of immune cells. Previous studies have shown that binding of IgG immune complexes to FcγRs on monocytes triggers potent inflammatory responses [42]. Additionally, Sabree et al., found that, monocytes were efficient at the phagocytosis of anti-Qβ-bound TLR9a VLPs and that the monocytes were stimulated through FcγR signaling rather than TLR9 agonism [28]. Therefore, it is possible that the monocyte activation observed in our studies with the TLRa VLPs may be due to the anti-Qβ-bound VLPs rather than TLR signaling.

Our studies demonstrated the critical role of APCs (specifically pDCs) in TLR7/8a VLP-induced T cell activation given the: 1 – lack of TLR7/8a VLP-induced T cell activation in isolated CD4 + and CD8 + T cells (Fig. 5B, C); 2 – the lack of TLR7/8a VLP-induced T cell activation when APCs (but not monocytes and NK cells) were depleted from PBMCs (Fig. 5D, E); 3 – the dependency of TLR7/8a VLP-induced T cell activation on pDCs (and not other pDC subsets) (Fig. 6); and 4 – the need for direct contact of T cells with pDCs for robust TLR7/8a VLP-induced T cell activation (Fig. 7). In support of this, previous work has shown that pDCs can establish stable contacts with T cells for an extended amount of time, forming functional immune synapses that are able to initiate T-cell activation [43]. Given the increased levels of IFNα and TNFα-positive pDCs induced by TLR7/8a VLPs compared to control, we sought to understand if IFNα in particular was responsible for T cell activation. However, we found that neutralization of TNFα (but not IFNα) partially but significantly suppressed TLR7/8a VLP-induced T cell activation (Fig. 4A, B). The partial suppression observed by TNF neutralization points to other cytokines being involved (such as IL-12 or IL-1) and we will follow up on these findings.

Lastly, we observed that the VLPs significantly increased levels of activated (CD69+) NK cells (Fig. 2F) and IFNγ-positive NK cells (Fig. 3F) compared to control. Previous studies have reported TLR7, TLR8 and TLR9 expression on NK cells which support our findings, although it is unclear whether NK cells have a functional TLR8 or not [44]. It appeared that depletion of APCs (but not monocytes) partially but significantly suppressed TLR7/8a VLP-mediated NK cell activation, but this suppression was not completely abrogated as observed with T cells (Fig. 3F). Additionally, neutralization of IFNα and TNFα had no effect on TLR7/8a VLP-mediated NK cell activation (Fig. 4C) suggesting the contribution of other cytokines or mediators and a potential direct effect of TLR7/8a VLPs with NK cells. Sabree et al., showed that fluorescently labeled TLR9a VLPs were taken up by NK cells only in the presence of anti-Qβ antibody, suggesting that the VLPs may be taken up by NK cells [28] by a FcyR-mediated mechanism. Nevertheless, the direct and/or indirect effects of TLR7/8a VLPs on NK cell activation imply its potential as an adjuvant for agents promoting NK cell activity and antibody-dependent cellular cytotoxicity (ADCC), such as cetuximab and trastuzumab. In fact, several preclinical studies including ours with TLR7/8 agonists have showed enhanced NK cell activity and ADCC in combination with cetuximab [6, 45, 46].

In this study, we used in vitro analysis of PBMCs to evaluate the anti-tumor immune response of TLRa VLPs. This approach offers several advantages, including direct human relevance, ready availability, and high viability in cell culture. However, it also has notable limitations. In vitro PBMCs lack the complex tumor microenvironment or in vivo trafficking; therefore, the immunosuppressive effects of tumor-associated macrophages and fibroblasts were absent. Moreover, the systemic immune response is not represented in PBMC cultures, which limits the ability to predict the clinical efficacy of candidate molecules. Despite these limitations, the data presented here provide important insights into the immune responses induced by TLR7a, TLR9a, and TLR7/8a encapsulated in VLPs, and suggests that TLR7/8a VLPs may be most efficacious in vivo at inducing an anti-tumor immune response. Nevertheless, in vivo evaluation is required to more fully understand memory T cell induction and anti-tumor immune responses in immunocompetent mouse models.

## Conclusions

**Fig. 8:**
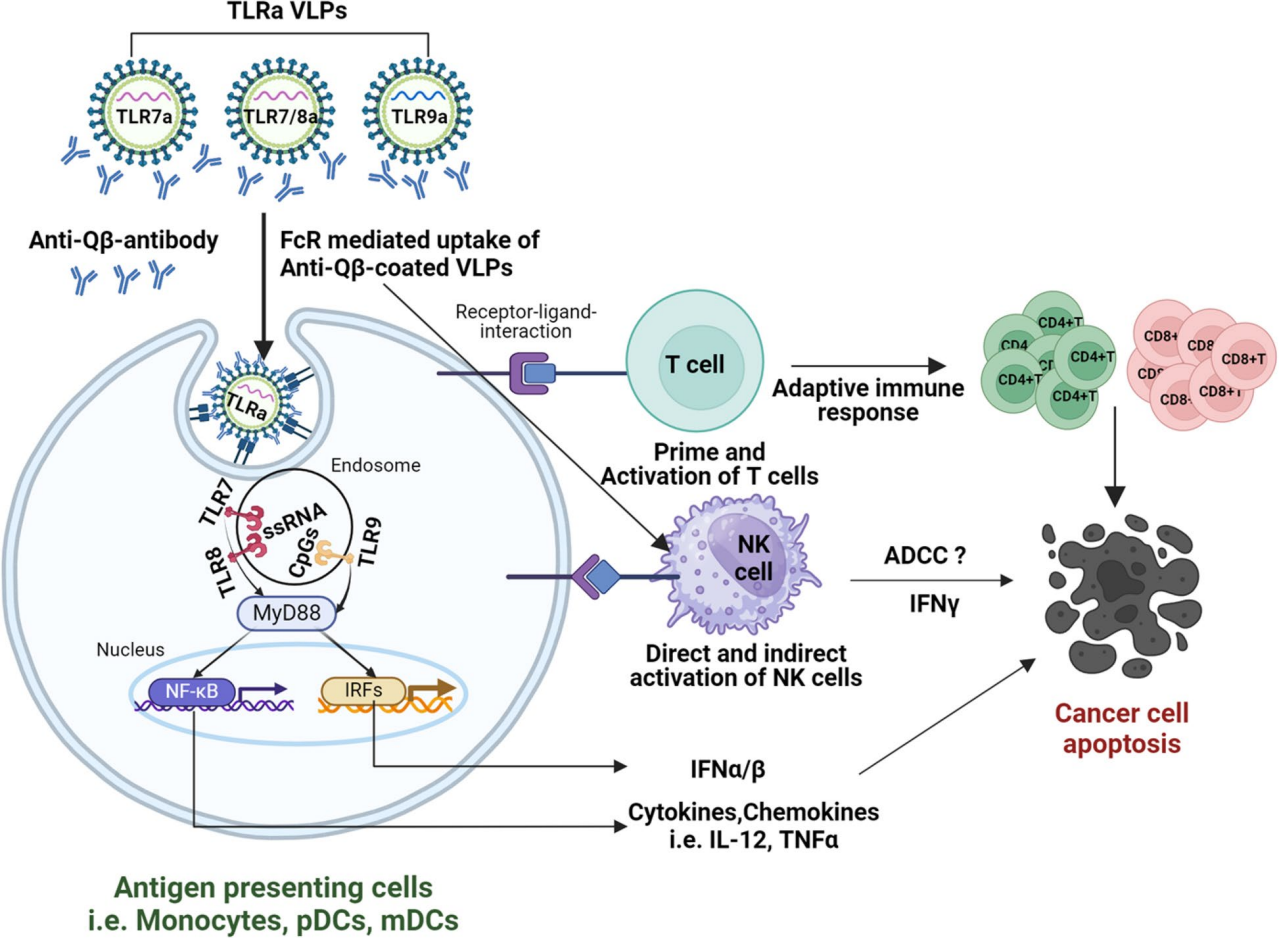
Graphical illustration of TLRa VLP-mediated immune cell activation and potential anti-tumor immune response

Overall, the results presented in this study shows that endosomal TLR agonists delivered through Qβ VLPs are an effective immunotherapeutic strategy for the activation of APCs (monocyte, pDC, mDC) leading to CD4 + and CD8 + T cell activation and potential anti-tumor immune responses (Fig. 8). The additional activation of NK cells by these VLPs are promising due to their ability to promote ADCC and enhance other immunotherapeutic agents (Fig. 8). The TLR7/8a VLPs triggered more robust immune responses compared to the TLR9a and TLR7a VLPs which is exciting given the ongoing positive results for TLR9a VLPs in combination with anti-PD1 therapy in clinical trials. The possibility that the TLR7/8a VLPs may elicit more potent anti-tumor immune responses compared to TLR9a VLPs warrants further investigation in preclinical and clinical trials as a novel immunotherapeutic strategy.

## Data Availability

The datasets generated and/or analyzed during the current study are available from the corresponding author on reasonable request.

## Abbreviations

TLR: Toll-like receptor
ODN: Oligodeoxynucleotide
PBMC: Peripheral blood mononuclear cells
VLP: Virus-like particle
NK: Natural killer
APC: Antigen presenting cell
DC: Dendritic cell
PDC: Plasmacytoid dendritic cell
MDC: Monocytic dendritic cells
IFNα: Interferon alpha
IFNγ: Interferon gamma
MYD88: Myeloid differentiation factor 88
ICS: Intracellular cytokine staining
ADCC: Antibody-dependent, cell-mediated cytotoxicity
